# Editorial: Plant-microbe interactions in heavy metal-contaminated environments

**DOI:** 10.3389/fpls.2026.1959855

**Published:** 2026-08-19

**Authors:** Zhongchuang Liu, Valentin Romanovski

**Affiliations:** 1 Department of Environmental Engineering Technology, College of Power Engineering, Chongqing Electric Power College, Chongqing, China; 2 Department of Materials Science and Engineering, University of Virginia, Charlottesville, VA, United States

**Keywords:** heavy metal contamination, microbial-assisted remediation, oxidative stress, phytoremediation, plant-microbe interactions, rhizosphere

Heavy metal pollution, due to its non-degradable nature and bioaccumulation effect, has become a global environmental problem that threatens the safety of ecosystems and the sustainable development of agriculture (Thalassinos et al., 2023; Campillo-Cora et al., 2025; Zhao et al., 2026; Xu et al., 2026). Long-term exposure to heavy metals can have adverse effects on human health (Liu et al., 2023; Campillo-Cora et al., 2025; Zeng et al., 2025; Yan et al., 2026). In the plant-microorganism system, heavy metals not only directly inhibit plant growth, hinder photosynthesis, and induce oxidative stress, but also profoundly alter the metabolic activities and community structure of soil microorganisms, thereby interfering with key ecological processes such as nutrient cycling and organic matter decomposition (Park et al., 2023; Huang et al., 2025). How to utilize the synergistic relationship between plants and microorganisms to remediate heavy metal-contaminated soil is not only a cutting-edge topic in basic ecology but also a key breakthrough point for environmental remediation technology innovation. In addition, the application of artificial intelligence in the treatment of heavy metal pollution is also worthy of attention (Gheibi et al., 2024; Da et al., 2027).

This Research Topic focuses on *“Plant-Microbe Interactions in Heavy Metal-Contaminated Environments”*, featuring five original research papers. These papers explore the multiple mechanisms by which the plant-microbe system responds to heavy metal stress from various perspectives, such as carbon allocation strategies, mycorrhizal symbiosis, biochar modification, rhizobium inoculation, and the application of nanomaterials. They provide an important theoretical basis and practical references for developing sustainable remediation strategies.

Lin et al. focused on the alpine meadow in the Sanjiangyuan area of the Qinghai-Xizang Plateau and investigated the regulatory effect of litter input on the carbon allocation strategies of plants and microorganisms. The study found that the influence of months on the carbon pool of the plant community and the microbial biomass carbon pool was significantly greater than that of grazing intensity. Among them, soil dehydrogenase and β-1,4-glucosidase jointly explained 27.31% of the variation in the carbon pool of the plant community. The results show the seasonal transformation law of carbon allocation strategies in alpine meadow ecosystem driven by carbon input, thus shedding light on how carbon cycling works in a grassland ecosystem under grazing disturbance. Xi et al. investigated the phytoremediation of vanadium-contaminated soil and concluded that arbuscular mycorrhizal fungus (AMF) such as *Rhizophagus irregularis* could potentially decouple the growth of *Setaria viridis* from vanadium accumulation. AMF inoculation increased the aboveground and root biomass of the plants by 109% and 104% respectively, while reducing the root vanadium concentration by 73% and the panicle vanadium concentration by 94%. Mechanistically, AMF achieved the effective separation of growth promotion and metal accumulation by inhibiting the enrichment and translocation of vanadium to the aboveground parts, altering the distribution of vanadium at the subcellular level (preferentially isolated in the cell wall), and upregulating chelators such as non-protein sulfhydryl and glutathione. This study laid an important foundation for the application of AMF in the biological remediation of vanadium-contaminated soil. Rajbanshi et al. conducted a methodical investigation of the influence of biochar amendment on the phytoextraction of twenty hazardous elements in coal fly ash polluted soil. The findings indicated that the introduction of coal fly ash resulted in a decrease in plant aboveground biomass as well as root biomass, but the use of biochar promoted the accumulation of plant biomass. The results of the principal component analysis demonstrated the clustering of legumes (*Vicia villosa* and *Lespedeza cuneata*) towards Ca and Mg clusters, whereas Gramineae (*Panicum virgatum* and *Festuca arundinacea*) clustered towards Al and Si clusters, which reflected a distinctive mechanism of absorption of different types of plants. This finding provides important guidance for the selection of plant species for the phytoremediation of coal fly ash contaminated sites. In addition, Pešić et al. explored the effect of rhizobium inoculation on the microbial community of alfalfa germinated in nickel-polluted soil. As a result, the researchers concluded that the use of rhizobium inoculation may lead to a potential increase of 38% in alfalfa production. Moreover, multivariate statistics showed that rhizobium inoculation, the heavy metal concentration in the soil and seasonal influences resulted in notable changes in microbial characteristics of the soil. This study proved that using a rhizobium-legume symbiosis could improve the growth of plants as well as the ecological state of the microorganisms of the rhizosphere in heavy metal stressed conditions. Furthermore, Chen et al. created a composite material of humic acid-containing nano-zero-valent iron (nZVI@HA) to deal with chromium and cadmium contamination. It turned out that the new material had an efficiency of extracting available chromium equal to 58.19%, and that for cadmium equal to 35.84%. Consequently, metals were exported to either the residual state, the biogenic-iron oxide state or the carbonate state by using chemical reduction, biosorption, and ion exchange. At the same time, nZVI@HA alleviated heavy metal stress and promoted microbial community diversity.

Together, these five studies present a multi-dimensional picture of the synergistic restoration of heavy metal pollution by plants and microorganisms. There are several aspects of carbon allocation strategies in grassland ecosystems, the decoupling of metal accumulation brought about by mycorrhizal fungi, the enhancement of the efficiency of plant extraction through the use of biochar, the change in the microecology of the soil environment brought about by the application of rhizobacteria, and the combined immobilization of metals presented by different types of composites. These types of research have broadened our knowledge of the interconnection among plants, microorganisms, and metals, providing us with the basis for effective strategies to remediate heavy metal pollution.
